# Severe liver injury with traumatic cardiac arrest successfully treated by damage control surgery and transcatheter arterial embolization in the hybrid operating room: a case report

**DOI:** 10.1186/s40792-021-01317-1

**Published:** 2021-10-30

**Authors:** Masahiro Hagiwara, Yoshihiro Iwata, Hiroyuki Takahashi, Koji Imai, Hideki Yokoo, Shunta Ishitoya, Miki Ogata, Naoto Matsuno, Yasuo Sumi, Hiroyuki Furukawa

**Affiliations:** 1 Division of Hepato-Biliary-Pancreatic and Transplant Surgery, Department of Surgery, Asahikawa Medical University, 2-1, Midorigaoka-Higashi, Asahikawa, Hokkaido Japan; 2 Division of Gastrointestinal Surgery, Department of Surgery, Asahikawa Medical University, Asahikawa, Japan; 3 Department of Radiology, Asahikawa Medical University, Asahikawa, Japan

**Keywords:** Blunt hepatic injury, Damage control surgery, Interventional radiology, Traumatic cardiac arrest, Hybrid operating room

## Abstract

**Background:**

The damage control approach is known to reduce the mortality rate in severely injured patients and has now become a common practice. Transcatheter arterial embolization (TAE) has been shown to be useful with combining with damage control laparotomy in identifying and controlling active arterial hemorrhage. Hybrid operating room (OR) allows both damaged control surgery and TAE in the same location in minimal time. We report a case of a patient with three cardiac arrests who was saved by early intervention using damage control surgery (DCS) with interventional radiology (IVR) in the hybrid OR.

**Case presentation:**

A 46-year-old woman was injured in a collision with a tree while snowboarding. She was eventually transported to hybrid operating room in our hospital with the diagnosis of significant liver laceration and hemorrhagic shock. Damage control surgery was performed with perihepatic packing (PHP) and TAE was conducted to stop active bleeding from right hepatic artery. She experienced 3 times of cardiopulmonary arrest, which was successfully resuscitated on each occasion. Although she had total of 3 times of laparotomy but tolerated well. She was discharged on day 82 of hospitalization and showed no neurological sequelae.

**Conclusion:**

Saving the life of a patient with severe trauma requires a multidisciplinary approach with cooperation and early information sharing among trauma team members. Sharing treatment strategy with the trauma team and early intervention using DCS with IVR in the hybrid operating room could save the patient’s life.

## Background

In the management of severe traumatic hemorrhage, mortality rate increases especially when traumatic cardia arrest is accompanied, and some authors have stated that attempted resuscitation from traumatic cardiac arrest is futile. However, understanding of the pathophysiology of traumatic cardiac arrest and advances in damage control resuscitation have led to unexpected survivors. The damage control approach is known to reduce the mortality rate in severely injured patients and has now become a common practice when patients develop the lethal triad of death [1, 2]. Active arterial hemorrhage could not be controlled by correction of coagulopathy, then it requires an adjunctive endovascular intervention procedure. TAE has been shown to be useful with combining with damage control laparotomy in identifying and controlling hepatic and splenic arterial hemorrhage. Hybrid OR allow damaged control surgery and TAE in the same location in minimal time. We report a case of a snowboarding collision patient with three cardiac arrests who was saved by early intervention using DCS with IVR in the hybrid operating room.

## Case presentation

**Fig. 1:**
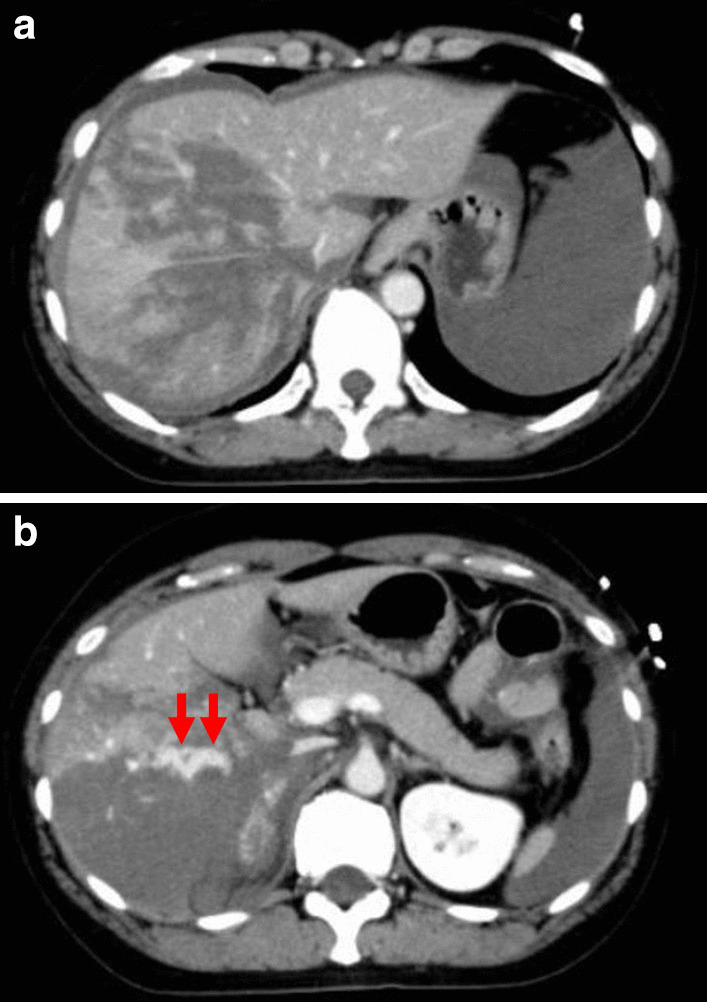
Abdominal CT scan taken at the local hospital prior to transportation to the University Hospital. **a** Irregular and mottled devascularized area occupied the whole right lobe of the liver. **b** Contrast extravasation from right hepatic artery (arrow) and hemoperitoneum

The patient was a 46-year-old woman. She was injured in collision with a tree while snowboarding. She was transported to the local hospital. On arrival, a vital sign showed a body temperature (BT) of 35.0 °C, blood pressure (BP) of 92/69 mmHg, heart rate (HR) of 81 bpm, respiratory rate (RR) of 30 breaths/min, Glasgow coma scale (GCS) score was 15. Focused assessment with sonography for trauma revealed intraperitoneal fluid collection. Contrast-enhanced computed tomography showed parenchymal disruption of the right hepatic lobe (grade IV) with extravasation of contrast material from the right hepatic artery to the peritoneal cavity (Fig. 1a, b), right renal contusion, fractures of the right 5th-8th ribs, traumatic subarachnoid hemorrhage, and acute subdural hematoma. Laboratory data showed that the serum hemoglobin was 8.7 g/dl, platelet count 173 × 10^3^/μl, and prothrombin time international normalized ratio 1.08, the fibrinogen level of 106 mg/dl. The metabolic acidosis extremely deteriorated with the base excess of − 15.08 mmol/l.

Despite of the fluid resuscitation with 1L of acetated Ringer's solution and 2 units of a type O uncrossmatched packed red blood cell, BP dropped to 70/45 mmHg and HR increased to 90 bpm. She was transferred to the university hospital for multidisciplinary treatment. Meanwhile she received a 6 units of type O uncrossmatched packed red cell transfusion. Just before the arrival, Although the airway was open, RR was decreased to 8 breaths/min, HR was elevated to 126 bpm, BP was unmeasurable, and GCS was scored as a 6. Her Injury Severity Score was 38, Revised Trauma Score was 3.19, and probability of survival was 26.0%.

**Fig. 2:**
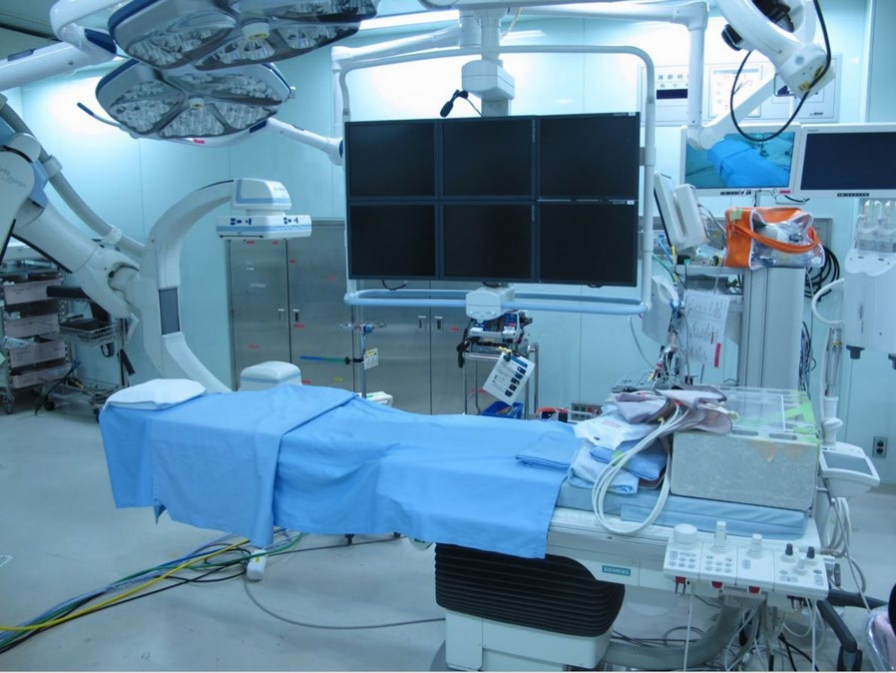
Hybrid operating room in our hospital. Artis zeegoⓇ is equipped for IVR

The trauma team was notified about conditions of this patient and hybrid OR equipped with Artis zeegoⓇ was set up (Fig. 2). Immediately after arrival, tracheal intubation and blood transfusion were performed in the primary care unit, and the patient was transferred to the hybrid OR within 10 min. She went into cardiopulmonary arrest in the operating room. Cardiopulmonary resuscitation was begun immediately, which resulted in a return of spontaneous circulation. At the start of surgery, her serum hemoglobin was 5.6 g/dl and platelet count was 2 × 103/μl, the prothrombin time international normalized ratio was 1.51, fibrinogen level 140 mg/dl, and base excess was -21.7 mmol/l.

A crash laparotomy was performed with a long midline incision which revealed a large laceration of the right lobe of the liver and intraperitoneal massive hemorrhage. Perihepatic packing (PHP) was conducted using the Pringle’s maneuver and the abdomen was left open with vacuum packing closure (VPC). However, bleeding was still not controlled. She went into cardiopulmonary arrest during the operation twice, but achieved a return of spontaneous circulation in each occasion.

**Fig. 3:**
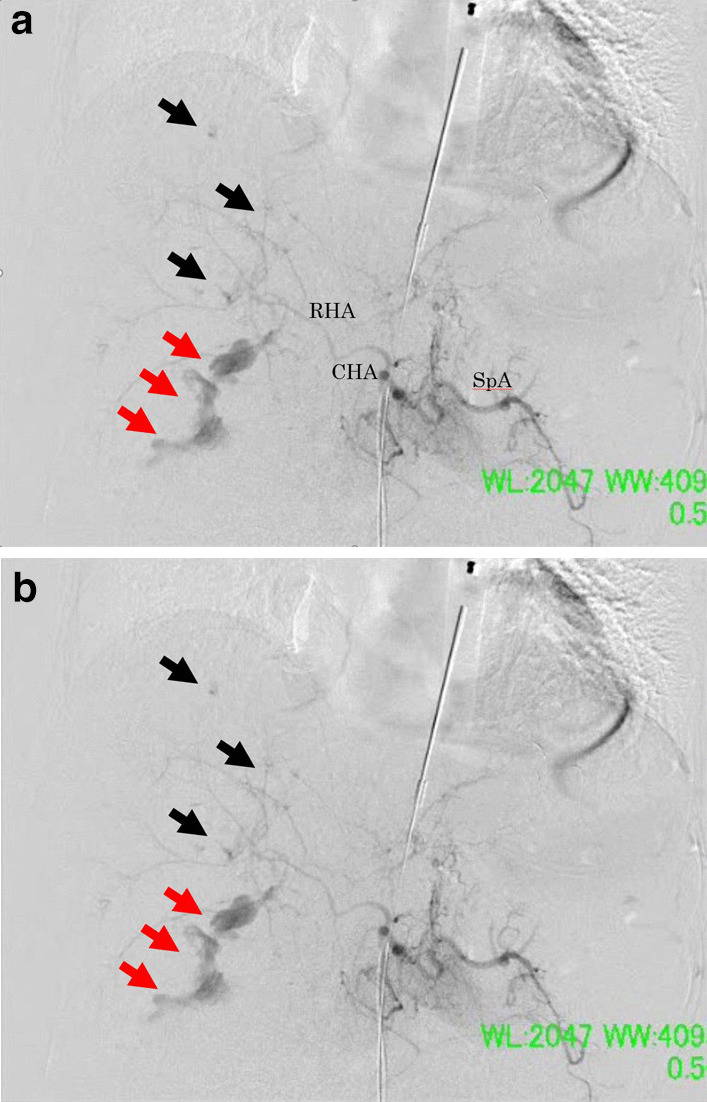
Hepatic angiography taken at the hybrid operating room immediately after perihepatic packing. **a** Contrast extravasation arising from the both anterior and posterior branches of the right hepatic artery (posterior branch: red arrows, anterrior branch: black arrows). *RHA* right hepatic artery, *CHA* common hepatic artery, *SpA* splenic artery. **b** Contrast extravasation disappeared after the both anterior and posterior branches were embolized with n-butyl cyanoacrylate

Transcatheter arterial embolization (TAE) was then performed by the radiologists. Supraceliac aortogram and selected hepatic arteriography were performed, which demonstrated contrast extravasation from the posterior and anterior branches of the right hepatic artery. Embolization of both arteries was performed with n-butyl cyanoacrylate (Fig. 3a, b). PHP with a towel wrapped in an iodine-impregnated surgical drape was performed. Hypothermia and acidosis worsened. The patient was transferred to the intensive care unit (ICU) with an open abdomen by VPC. Operation time was 120 min; 27 min for surgery, 93 min for TAE. The intraoperative blood loss was 16,400 ml, and the intraoperative transfusion included 76 units of packed red blood cells, 60 units of freshly frozen plasma, and 40 units of platelets.

On admission to the ICU, her BP was 72/51 mmHg, HR was 109 bpm, and body temperature was 32.1 °C; her fibrinogen was 140 mg/dl, and base excess was − 19.1 mmol/l. The hemodynamic state was gradually improved with restoration of physiological function including rewarming, and correction of coagulopathy and acidosis in the ICU. However, bleeding from the liver laceration was found at the second-look laparotomy performed 18 h after the first surgery. PHP with VPC was repeated and she was transferred back to the ICU. After further resuscitation in the ICU, a third operation was performed 46 h after the second operation. When towel packing was removed, no further bleeding was detected. Gallbladder necrosis was found, and cholecystectomy was performed. After the hemostasis was secured, the abdomen was completely closed. Tracheotomy was performed on day 7 after admission. She was discharged from the ICU on day 10 after admission. She was weaned off from the ventilator on day 16 after admission. She was placed with an endoscopic nasobiliary drainage tube for biloma on day 27 after admission, which was removed on day 66 after admission. She was discharged on day 82 after hospital admission and alive and no neurological sequelae.

**Fig. 4:**
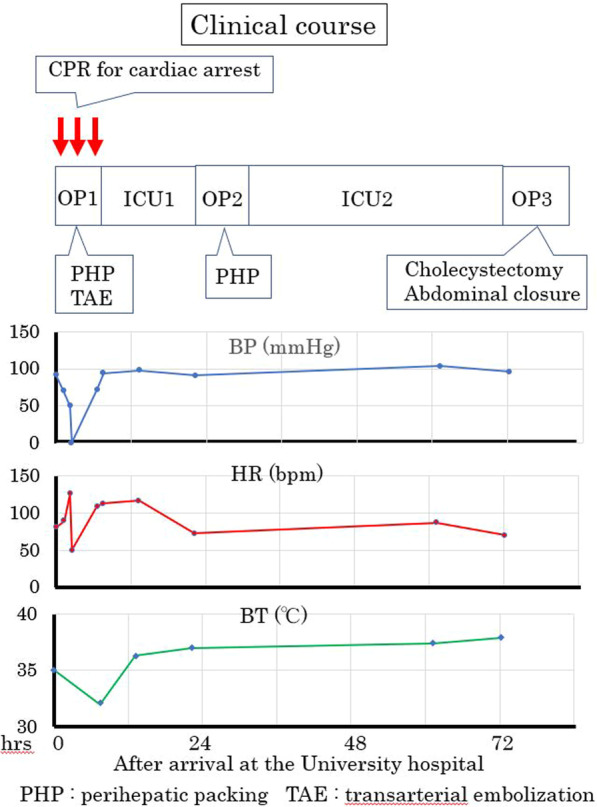
Clinical course first 72 h after arrival at the University Hospital.Treatments performed during each event were shown as below; OP1: Damage control surgery performed with perihepatic packings, TAE for active bleeding from the both anterior and posterior branches of the right hepatic artery, vacuum packing closure (VPC). OP2: PHP and VPC was repeated. OP3: cholecystectomy for the gallbladder necrosis, complete abdominal closure ICU1 and ICU2: correction of hypothermia, acidosis, and hypotension

The clinical course is shown in Fig. 4.

## Discussion

The survival rate for patients with out-of-hospital cardiac arrest due to trauma is extremely low [3, 4]. The overall survival rate for traumatic cardiac arrest has been reported to be 5.6 and 1.6% for cases with a good neurological outcome. The respective survival rates for traumatic cardiac arrest after blunt and penetrating trauma have been reported to be 3.1 and 3.3% respectively, and 1 and 1.9% for cases with a good neurological outcome, and the prognosis for traumatic cardiac arrest due to blunt and penetrating trauma is generally recognized as even poorer [5].

Although it is becoming a dominant view that a patient with traumatic cardiac arrest is futile, the present report describes a case in which the patient survived three cardiac arrests without neurological deficits. There were two reasons to accomplish successful lifesaving of this patient; one was an availability of hybrid OR where both the damage control laparotomy and TAE were possible on the same table, the other was that trauma team cooperate with sharing the information of the patient even prior to her arrival.

**Fig. 5:**
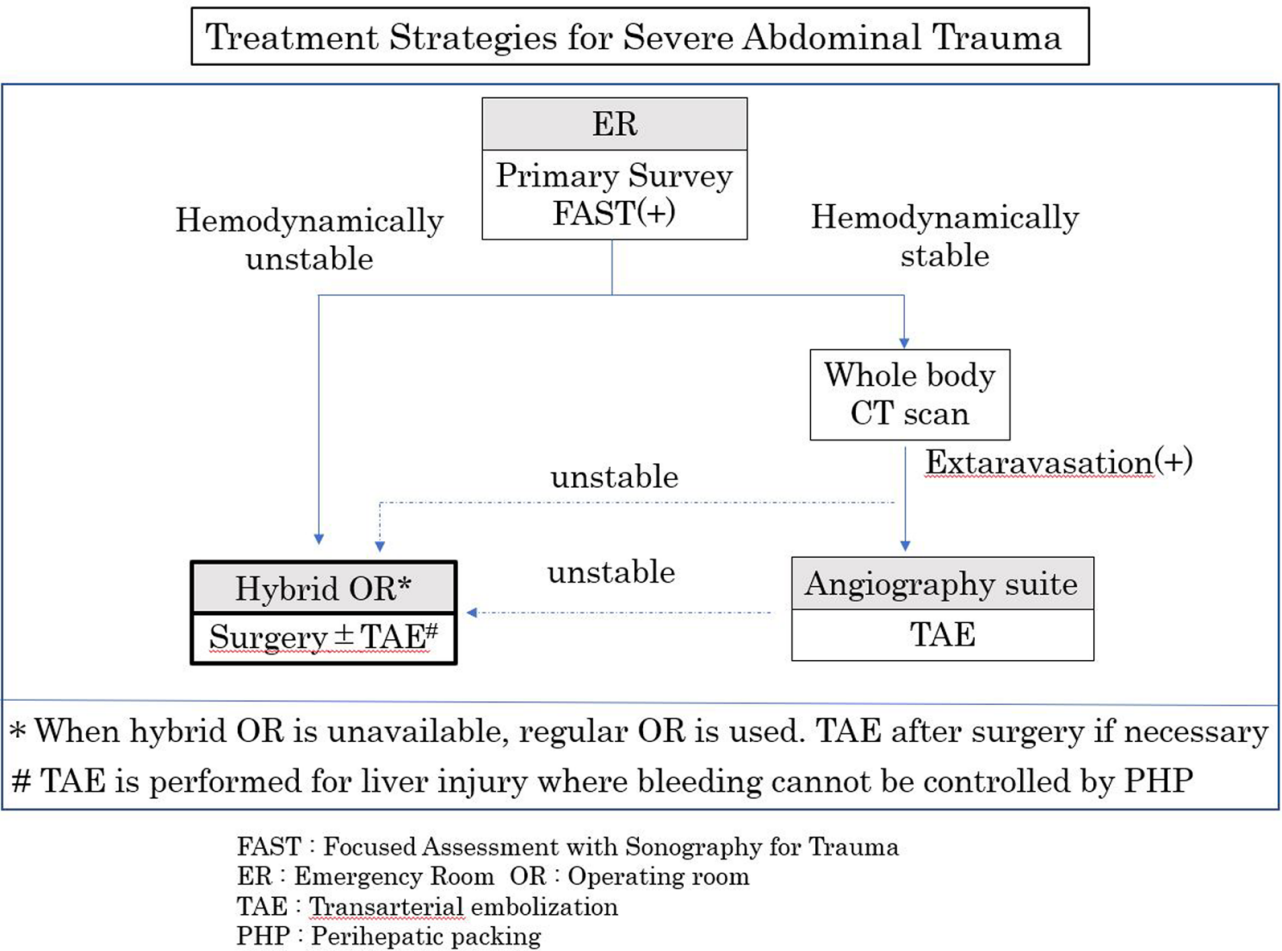
The treatment strategies for severe abdominal trauma at our hospital.When a severely injured patient arrives at the ER. FAST was performed. If the patient is hemodynamically unstable, he is transferred directly to hybrid operating room to perform surgery and /or IVR (TAE). In case hybrid OR is not available, he is operated at regular OR and go to angiography suite if necessary. If the patient is stable enough, he goes to CT scan. If there is contrast extravasation he goes to angiography suite to obtain IVR (TAE). If there is no contrast extravasation he is observed in ER or ICU. In anytime the patient become unstable he is going to have surgery at the either hybrid or regular OR

Damage control laparotomy had been generally accepted as standard strategy with a reduction in mortality in severely injured patients [6–9]. With damage control laparotomy, hepatic hemorrhage can be controlled by perihepatic packing initially followed by correction of hypothermia, acidosis, and coagulopathy in the ICU, and re-operation with definitive repair of hepatic injuries later. However, the pressure created by adequate PHP can control only portal and hepatic venous hemorrhage, but not hepatic arterial hemorrhage. When active arterial hemorrhage exists, it requires additional procedures such as TAE to stop the ongoing hemorrhage. TAE is reportedly effective in controlling arterial bleeding after DCS in patients with unstable circulatory dynamics [10–14]. Surgery and IVR are complementary in the treatment of patients with severe trauma. In the present case, TAE was performed because bleeding continued in spite of PHP. Active bleeding stopped by embolizing the anterior and posterior branches of right hepatic artery. The treatment strategies for severe abdominal trauma at our hospital is shown in Fig. 5.

As a primitive hybrid OR, “RAPTOR (Resuscitation with Angiography, Percutaneous technique, and Operative Repair) suite” became available in 2011 by the trauma center in Calgary, Canada and Sydney, Australia with the concept that emergency percutaneous therapies, open surgery, as well as resuscitation and critical care should be performed in the same physical location. Combining multiple functions in one location, saves time moving patients from one venue to another. In the past, when we combined DCS and IVR in the treatment of patients with severe trauma, we had to move the patient from the operating room to the angiography suite. An availability of the hybrid OR in the present case saved travel time and allowed us to perform rapid hemostasis. Thus, the use of hybrid OR markedly reduces the time required for surgery and IVR, and have the potential to improve the survival rate of patients with multiple traumatic injuries [15–17].

Hybrid OR are becoming more popular worldwide. However, clinical outcome and efficacies of hybrid OR have not been elucidated until recently. There were 2 articles reporting clinical outcome and efficacies of the hybrid OR in a year of 2020 [18, 19]. One was the article Caver rt al. reported. One hundred and sixty-nine patients who were transferred to the hybrid suite were compared with the same numbers of historical control. Compared with the historical control, use of the hybrid suite resulted in shorter arrival to intervention (82 min vs 148 min (angiography suite) and 101 min (operating theater), *p* < 0.05) and a clear benefit of for survival was evident (42 vs 22%). The other was that Loftus reported 186 cases who underwent surgery in the hybrid OR. The interval between OR arrival and hemorrhage control was shorter in the hybrid cohort (49 vs 60 min, *p* = 0.005). The hybrid cohort had fewer infectious complications (15 vs 27%, *p* = 0.009) and ventilator days (2.0 vs 3.0, *p* = 0.011) compared to the control, and similar in-hospital mortality (13 vs 10%, *p* = 0.579). Both articles evaluated the outcome of total patients using the hybrid OR and the efficacies of the hybrid OR was apparent. Considering that the real value of the hybrid OR is when it is used for both surgery and IVR same as the present case, further analyses will be expected to confirm it.

Since the cardiac arrest occurred in the OR, the patient was able to be resuscitated quickly and was saved despite of three cardiac arrests. The trauma team including the surgeons, radiologists, anesthesiologist, emergency physicians and operating room nurses at the university hospital was notified about conditions of this patient and hybrid OR was set up. The speed and accuracy of the decision-making process has a great impact on the patient's survival and functional outcome. In addition, the efforts of the blood transfusion department in collecting blood products may have contributed to the patient's survival. We believe that the patient survived and was discharged from the hospital with a good neurological outcome due to the care of the medical team in cooperation with other departments and professionals with a good understanding of the concept of damage control resuscitation.

## Conclusion

To save the life of a patient with severe trauma, it is important to adopt a multidisciplinary approach through cooperation and early information sharing with each department (surgery, emergency, radiology, anesthesiology). In the present case, the early involvement of the radiology department and the use of the hybrid OR are considered to have saved the patient's life. Sharing of strategies, such as blood transfusion, TAE, a trauma team approach to surgery, and the early decision to perform DCS may improve the outcome of patients with severe abdominal trauma.

Hybrid treatment that combines emergency surgery and intraoperative IVR provides a prompt and appropriate management approach for the treatment of patients with severe liver injury and may improve patient outcomes due to the reduction in the treatment time.

## Data Availability

The relevant data and images related to the patient’s course and care are included in the article.

## Abbreviations

TAE: Transcatheter arterial embolization
OR: Operating room
DCS: Damage control surgery
IVR: Interventional radiology
PHP: Perihepatic packing
VPC: Vacuum packing closure
ICU: Intensive care unit
